# Liquid gating membrane

**DOI:** 10.1093/nsr/nwz197

**Published:** 2019-11-28

**Authors:** Xu Hou

**Affiliations:** College of Chemistry and Chemical Engineering & College of Physical Science and Technology & Collaborative Innovation Center of Chemistry for Energy Materials & State Key Laboratory of Physical Chemistry of Solid Surfaces, Xiamen University, China

**Figure 1.**
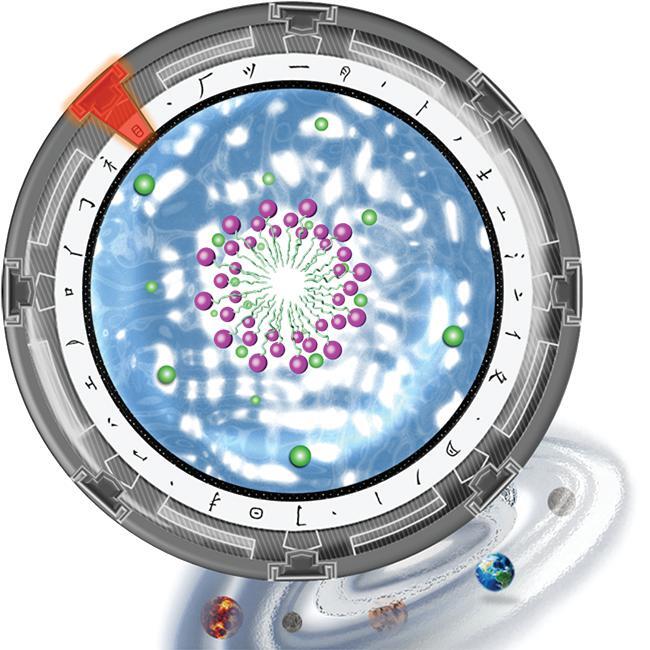
The notion of a liquid-state ‘Stargate’ in the science fiction movie—a kind of liquid ‘door’ that distorts time and space—inspires us to ask whether liquids can indeed be used as gates. Inspired by *Stargate*, the figure shows the liquid gate open and closed, which can be designed just as ‘address-dialing’ is in *Stargate*. As each symbol is dialed in the correct order, the Stargate is open. Here I design the symbols using Chinese characters; with the right order, we can get ‘响应性液体门控’ as the main concept of a responsive liquid gate expressed in Chinese.

The development of structural materials has brought impetus to every industrial revolution. The value and uses of structural materials follow primarily from their mechanical properties rather than their optical, electronic, magnetic or chemical properties. Conventionally thinking, structural materials are solid, with a fixed structure and good stability, which could include the materials response to an applied force. However, the notion of a liquid-state ‘Stargate’ in the 1994 science fiction movie—a kind of fluid ‘door’ that distorts time and space [1]—inspires us to ask whether liquids can indeed be used as structural materials, such as responsive liquid gates (Fig. 1).

At the macroscale, the answer is no: a liquid has too much mobility, and its molecular interactions are not as strong as those in solids. It has no inherent shape that can form a stable structural material. At the microscale, however, the situation is completely different. In that case a liquid can be stable if confined in space by capillary forces, albeit while being reconfigurable using applied forces. Then the liquid can be used as a stable structural material with new functions and applications [2].

Next, membrane-based materials are selected as a representative example to discuss the possibility of using liquid as a structural material and its bright application prospects. Membrane science plays a central role in fields ranging from desalination to medicine, but still current studies are limited in handling the complex sorting and multiphase substances needed in numerous real-world applications [3]. Designing gated micro/nanopores of membranes that can responsively sort and transport complex multiphase substances is essential for areas as diverse as gas, oil, wastewater, blood processing, liquid and aerosol therapies, 3D-printing technology, refreshable tactile displays, soft robotics, organs-on-a-chip and other micro/nanofluidic systems. But, in spite of the progress made in programming specific gating and transport behaviors, a single system capable of complex multiphase control and selectivity is still a distant prospect, and fouling is nearly inevitable. The trade-offs between surface chemistry and size requirements make it difficult to differentially tune the behaviors of gases and liquids simultaneously, and selectively allowing liquids while blocking gas seems like mission impossible. The material requirements of responsive gates can further narrow down the options, as can efforts to minimize fouling. Addressing these challenges requires new membrane materials that can reconcile these often inherently competing demands.

**Figure 2.**
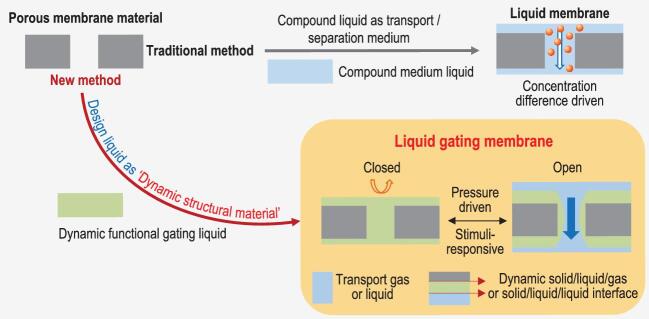
The difference between a liquid membrane and a liquid gating membrane. For the traditional method, the compound medium liquid is used to add internal porous membranes to prepare liquid membranes driven by the concentration difference. For the new method, to design dynamic functional liquids as dynamic structural materials on a pressure-driven basis, the liquid gating membrane can use a capillary-stabilized functional gating liquid to form reversible gates inside the porous membranes.

The liquid gating membrane was first proposed in 2015 [2]. Distinct from liquid membranes, which are based on the chemical potential driving mechanism [4], the liquid gating membrane as a structural material is the liquid-based membrane materials response to an applied force as a pressure-driven system, which uses a capillary-stabilized functional liquid to form reversible gates inside the pores, showing prominent properties in controlling complex, selective, multiphase substance transport (Fig. 2). The difference between liquid gating membranes with other structural materials is mainly based on their liquid properties, including the benefits of lower transmembrane pressure, tunable multiphase selectivity, mobility and incompressibility, non-covalent binding, defect-free material interface, molecular scale ultra-smooth surface, self-recovery, self-adaption, anti-fouling, anti-icing, etc. The current frequently-used pore sizes of liquid gating membranes are at the microscale, because polymer porous materials are mostly used, and if we want to use a smaller pore size, it needs porous materials with higher mechanical properties, such as ceramics, metals or composites. Until now, liquid gating membranes for filtration applications that can selectively process complex material flows, precisely separating liquids, gases, and solids without clogging and with significant energy savings, is in the process of real business promotion [5]. To convert the existing standard membrane filters to liquid gating membranes, the key point is to design the functional gating liquid to have affinities with the existing standard filters.

It is worth mentioning that the liquid gating membrane intrinsically generates differential response profiles for a broad spectrum of gases and liquids over a wide range of environmental pressures—in one system—with virtually no fouling for complex fluids. Rather than reconfiguring the solid geometry or relying on molecular switches or external gates, this liquid gating membrane takes advantage of the unique ability of a fluid to deform and reconfigure *in situ* to respond to capillary pressure. However, without responsive interface design it would greatly limit the development and application of the liquid gating membranes, since gating depends on both mechanics and chemistry in the form of interfacial tension. If there is a responsive system with tunable pore sizes of solid membranes and responsive gating liquids, the gating membrane will open numerous opportunities for energy-efficient, complex multiphase sorting in areas ranging from fuel harvesting, water treatment, and biomedical fluid processing to 3D-printing technology and soft robotics to microscale reactors and microchip sensors.

Based on the physicochemical designs of responsive interface, smart liquid gating membranes have been put forward [3, 6]. The responsive liquid gating system represents a new regime in the study to transform the basic scientific issues of the membranes from the solid–liquid/solid–gas interface to the liquid–liquid/liquid–gas interface for bringing more possibilities to the design of smart functional systems. To further illustrate this, the liquid-constructed interface is molecularly smooth, and the liquid can transform constantly to adapt to the external environment, which makes it a good desired dynamic structural material to open and close solid membranes. Therefore, it would bring many new potential applications, which could not be realized in traditional membrane systems.

For example, in traditional systems, most of the multiphase membrane separation is based on applied pressure changes. Firstly, degassing by reducing the pressure [7] or liquid–liquid separation by increasing the pressure [8] often need extra energy to let the applied pressure change. Concurrently, the applied pressure changes will also influence the entire separation system, not just the membrane itself, and thus the energy acting on the other parts is wasted. Secondly, the instable pressure conditions may affect numerous real-world applications, because many chemical reactions require steady-state pressure conditions to guarantee high chemical efficiency and yield. However, the liquid gating elastomeric porous membrane can be used to achieve tunable pore deformation without altering the applied pressure [9]. Therefore, it will bring new opportunities for numerous membrane separation applications under constant pressure, such as gas-involved chemical reactions, multiphase separation, fuel cells, multiphase microreactors, etc.

In another example, the chemical detection of a certain substance is so vital that new detection mechanisms with features such as low cost, accessibility, and readily available visual markers are in demand. A liquid gating membrane-based chemical detection technology has been developed, which has a dynamic gas–liquid interface due to dipole-induced interfacial molecular reconfiguration [10]. The technology shows a sensitive relationship between the liquid gating behavior and the dipole-force-induced rearrangement of interfacial molecules. The above features could be used to create visual markers for detection by converting the analyte-mediated interfacial interaction to a pressure-driven marker movement. This liquid gating detection approach needs no electrical energy input and has readily available markers for anyone to see directly. This new approach opens a window for a more in-depth exploration of combining liquid gating membranes with chemical detection mechanisms.

Liquid gating membranes are still in their early stages. They will be greatly enhanced by the development of both responsive solid porous membranes and functional gating liquids that are capable of producing more smart functional liquid gating membrane systems. In this case, liquids can be used as stable structural material, bringing dynamic time response, excellent fouling resistance and a drag reduction effect at a molecular level without defects at any time. A breakthrough in many new applications, such as membrane separation, chemical detection, water treatment, air purification, high-efficiency catalysis, battery separators, microfluidics, 4D printing, etc. is therefore expected from implementing responsive liquid gating membranes, an area that is still developing.
